# Ligand-dependent localization and intracellular stability of sigma-1 receptors in CHO-K1 cells

**DOI:** 10.1186/1750-2187-2-8

**Published:** 2007-09-20

**Authors:** Timur A Mavlyutov, Arnold E Ruoho

**Affiliations:** 1 Department of Pharmacology, University of Wisconsin, School of Medicine and Public Health; 1300 University Ave, Madison, WI 53706, USA

## Abstract

**Background:**

Sigma-1 receptors are involved in regulation of neuronal activities presumably through regulation of the activity of ion channels. Sigma-1 receptors also play a role in growth and metastasis of cancerous cells. Intracellular distribution of sigma-1 receptors have been linked to sphingolipid-enriched domains.

**Results:**

We report that in CHO-K1 cells sigma-1 receptors target to focal adhesion contacts (FAC) where they colocalize with Talin and Kv1.4 potassium channels. The levels of sigma-1 receptors in the FAC were significantly increased by application of sigma-1 receptor ligands and by filamentous actin (F-actin) polymerization with phalloidin. The total length of FAC (measured by the focal adhesion marker, talin) was concomitantly increased in the presence of sigma-1 receptors upon phalloidin treatment. Only sigma-1 receptor ligands, however, resulted in an increase of sigma-1 receptors in the FAC, independent of talin. Additionally, a novel approach was utilized to allow an assessment of the half life of endogenous sigma-1 receptors in CHO-K1 cells, which was measured to be at least 72 hours.

**Conclusion:**

Ligand activated sigma-1 receptors translocate into FAC from a pool of receptors stored in ER lipid rafts presumably for inhibition of Kv1.4 channels. Stabilization of actin filaments is likely to be important for targeting sigma-1 receptors to Focal Adhesion Contacts in CHO-K1 cells.

## Background

Sigma-1 receptors have been shown to be expressed in many tissues and enriched in the brain[1]. This receptor was first described as a type of opioid receptor[2], but further experiments showed that the sigma-1 receptor has distinct pharmacological properties[3]. It binds to drugs such as (+)-pentazocine[4], cocaine[5], methamphetamine[6]) and neurosteroids[7] and is implicated in certain forms of psychoses[8], pain tolerance[9], learning and memory and attention deficit disorders[8]. Myriad properties that are associated with sigma-1 receptors led some to consider the receptors as intracellular amplifiers[10]. Endogenous ligands are unknown at present and common signal transduction pathways remain elusive.

However, electrophysiological experiments have shed some light on a significant role of sigma-1 receptors involving an inhibitory action on the activity of ion channels such as potassium Kv1.4, Kv1.5[11] and chloride VRCC[12] channels. These data provided evidence for a possible general function of sigma-1 receptors in maintenance of the excitability of cell membranes in response to psychotomimetic drugs. Involvement of sigma-1 receptors in a plethora of known pathways is implied in it's gene promoter, which contains an AP-1 complex (regulating immediate early gene products) binding sequence[13]. Inhibition of AP-1 expression through suppression of the extracellular receptor kinase (ERK) MAPK pathway activity resulted in significant downregulation of the total level of sigma-1 receptors[14]. On the other hand, activation of sigma-1 receptors resulted in upregulation of the FRA-2 gene[15,16], a member of the AP-1 subfamily.

Sigma-1 receptors have been predicted to be transmembrane proteins[11]. They are localized to sphingosine-enriched lipid rafts on the bundles of the endoplasmic reticulum (ER) and have been shown to target to the plasma membrane (PM) as well. Translocation of sigma-1 receptors from the ER to the PM has been reported[17,18]. In cultured oligodendrocytes sigma-1 receptors are involved in trafficking of galactosylceramide, an important component of myelin[19].

Additionally, cancer cell lines have been shown to be significantly enriched with sigma-1 receptors[20]. Application of sigma-1 receptor drugs as well as silencing of sigma-1 receptors with RNAi inhibited cell adhesion to the plating surface and suggested novel functions of sigma-1 receptors in metastasis[20].

Here we report for the first time that in Chinese hamster ovary (CHO-K1) cells sigma-1 receptors target to focal adhesion contacts (FAC) where they colocalize with Kv1.4 and talin. The appearance of sigma-1 receptors in the FAC increased significantly upon treatment with the sigma-1 receptor ligands (+)-pentazocine, haloperidol and iodoazidococaine[21,22] as well as by stabilization of filamentous actin with phalloidin. We also utilized a novel protocol for photolabeling endogenous sigma-1 receptors in live cells, allowing us to determine a significant intracellular stability of the endogenous receptor pools in CHO-K1 cells.

## Results

**Figure 1:**
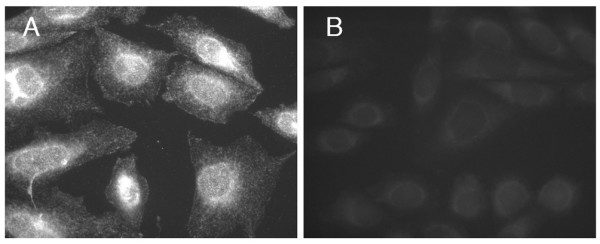
**Preadsorbtion control showing the specificity of the Anti-sigma-1 receptor antibody**. CHO-K1 cells stained with anti-sigma1 antibody (0.6 μg/ml). A – no preadsorbtion; B – before immunostaining antibody was preadsorbed for 1 h with the purified sigma-1 protein (25 μg/ml).

**Figure 2:**
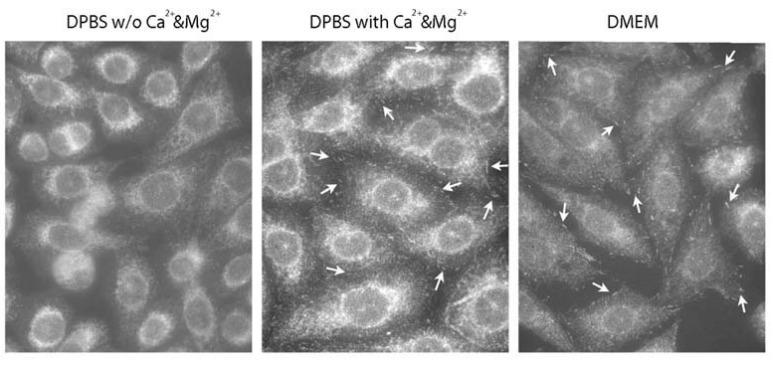
**In CHO-K1 cells during a 30 sec rinsing with buffers with or w/o Ca^2+ ^and Mg^2+ ^before fixation changed the intracellular distribution of endogenous sigma-1 receptors**. A. Cells were rinsed with DPBS w/o Ca^2+ ^and Mg^2+ ^and DMEM. B. Cells were rinsed with DPBS with different concentrations of Ca^2+^ and Mg^2+^

The specificity of rabbit polyclonal antibody against the purified whole-sequence sigma-1 receptor protein[23] was confirmed by preadsorbtion with antigen (Fig. 1). The sigma-1 receptor immunocytochemical staining revealed that the receptors were localized to the nuclear envelope (NE), endoplasmic reticulum (ER) and the focal adhesion contacts (FAC). In order to detect sigma-1 receptors in the FAC, it was critical, before fixation, to rinse the cells for a short   time (30 sec) with DMEM (no serum present) or DPBS with Ca^2+^ & Mg^2+^ present (Fig. 2.). These results support the present literature data[17,18] indicating that the sigma-1 receptors are able to alter their distribution within the cell and further indicates that divalent cations can modify this distribution, especially in the FAC.

**Figure 3:**
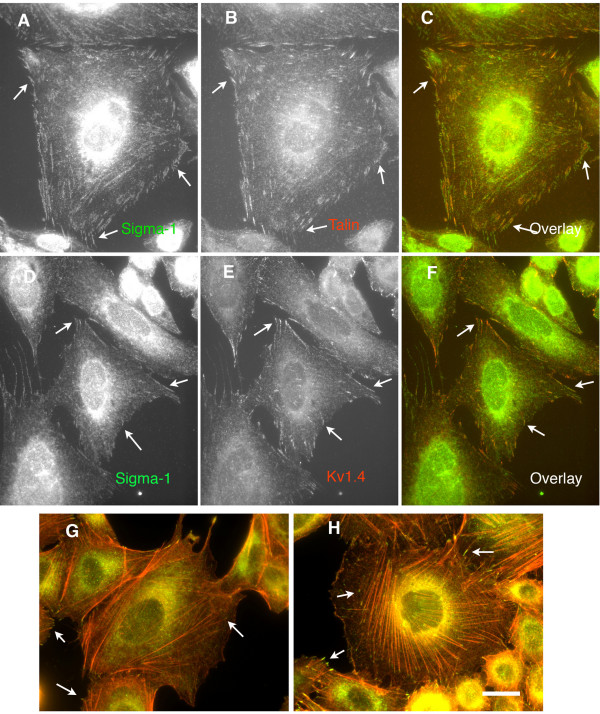
**Endogenous Sigma-1 receptors colocalize with other proteins in the FAC**. A-C. Colocalization with talin. A-sigma-1; B-talin; C-Overlay. D-F. Colocalization with Kv1.4. potassium channel. D-sigma-1; E-Kv1.4.; F-Overlay. G, H. Colocalization of sigma-1(green) and F-Actin(red) in mock or phalloidin (50 ng/ml for 30 min) treated cells. Note, that the sigma-1 localized on the the barbed ends of actin filaments.

To support the observation that the sigma-1 receptors are present in true FAC a double stain for talin (a FAC marker) was used to show a high level of colocalization (Fig. 3. A–C). It has been previously demonstrated [11,24,25], that sigma-1 receptors modulate the activity of Kv1.4 potassium channels. We found that in CHO-K1 cells Kv1.4 is also targeted to FAC under all sample preparation conditions and double staining for Kv1.4 and sigma-1 receptors showed colocalization in the FAC (Fig 3. D–F).

Further, we show that the sigma-1 receptors were found to localize to the tips of F-Actin rods, where the FAC form (Fig 3. G,H).

**Figure 4:**
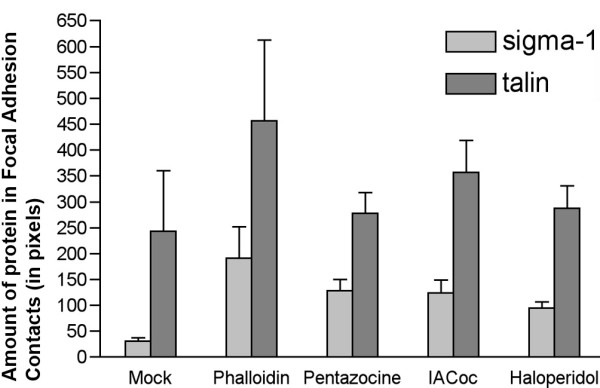
**Dynamics of endogenous Sigma-1 receptor and Talin appearence in the FAC in CHO-K1 cells under various treatment conditions**. All the compounds indicated were added to cells in complete 10% FBS supplemented DMEM for 30 min at a concentration of 10 μM except phalloidin (50 nM). Morphometrical quantitation of the amount of the sigma-1 receptor or talin in the FAC was performed with the Axiovision 4.3 software. The total amount of staining (in pixels) for each channel was quantitated for each image and divided by the number of cells (detected by DAPI staining of nuclei). 101–155 cells were measured per each condition.

Intracellular protein-protein interactions of the sigma-1 receptors in a response to stimulation by specific ligands such as (+)-pentazocine, SKF10047 or cocaine has been demonstrated to change[18]. We therefore explored the possibility that various sigma-1 receptor ligands could alter the amount of receptor in FAC. In CHO-K1 cells following various treatment with (+)-pentazocine, iodoazidococaine and phalloidin (which directly stabilizes F-Actin) we observed that there was a significant increase of the level of sigma-1 receptors in the FAC (Fig. 4.). The level of talin in the FAC in a response to the various ligands showed only a slight increase. Following direct actin filament stabilization with phalloidin a doubling of the level of talin in the FAC was observed, while the sigma-1 receptor levels in the FAC increased more than 6 fold (Fig. 4.).

**Figure 5:**
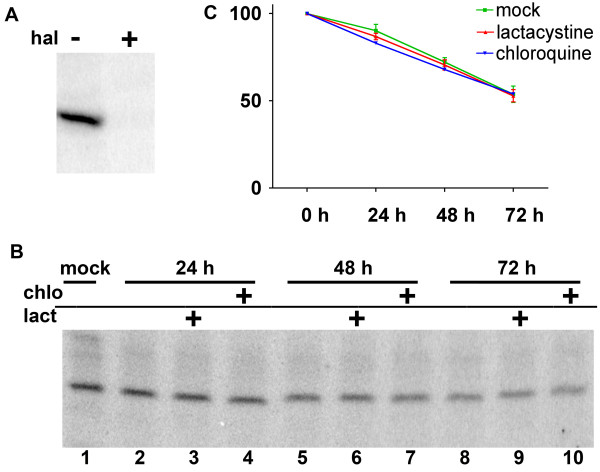
**Stability of the sigma-1 receptors in CHO-K1 cells**. Endogenous sigma-1 receptors were [^125^I]-IACoc photolabeled in live CHO-K1 cells by a brief photolysis (see Materials and Methods for details). Labeled cells were washed free of excess ligand and plated on culture dishes for various periods of time with or without inhibitors of proteolysis. A. Specificity of I^125^-azidococaine labeling in live cells was tested by preincubation of cells with 5 μM haloperidol (hal), prior to [^125^I]-IACoc labeling (plus conditions). B. Autoradiogram of CHO-K1 cell lysate collected at different time points after plating of photolabeled cells: time 0 – (lane # 1); after 24 h – (lanes # 2,3,4); after 48 h – (lanes # 5,6,7); after 72 h – (lanes # 8,9,10). The proteosomal inhibitor, lactacystine, 2 μM (lanes #3,6,9) was added; the lysosomal inhibitor, chloroquine, 100 nM (lanes # 4,7,10) was added. C. Quantified data from the situ photolabeled sigma-1 receptor time-course from 3 independent experiments are represented in part B. These data indicate that in CHO-K1 cells the turnover of the ligand occupied sigma-1 receptor is slow with a half-life at least 72 hours.

Since the increase in sigma-1 receptors in FAC upon addition of sigma-1 receptor ligands occured in a relatively short period of time (30 min), we hypothesized that this relocation may be due to recruitment of receptors from intracellular pools. In order to determine the availability of the sigma-1 receptors for translocation, we assessed the stability of the receptors in CHO-K1 cells. These experiments were performed by using a unique intracellular photolabeling approach with the high affinity sigma-1 receptor photoprobe, [^125^I]-IACoc, which forms a covalent linkage with the receptor residues upon photolysis[21,22]. Fig. 5 shows that *in situ *specific photolabeling in live cells was successful in identifying the sigma-1 receptor. Application of this "pulse-chase" approach for intact cell labeling therefore provided an opportunity to address the question of intracellular stability of photolabeled sigma-1 receptors thus demonstrating that a considerable stable intracellular pool of sigma-1 receptors occurs in CHO-K1 cells. Potential mechanisms of degradation or turnover of the sigma-1 receptors using inhibitors of proteosomal and lysosomal protein digestion were further investigated. The results demonstrated that the half life of the ligand-bound form of the sigma-1 receptor in CHO-K1 cells is 72 hours and was insensitive to lysosomal (chloroquine) and proteosomal (lactacystine) inhibitors.

## Discussion

The data reported in this paper show a profound diversity in sigma-1 receptor distribution in CHO-K1 cells. Sigma-1 receptors have previously been shown to localize to the plasma membrane[11,20,26]. In contrast to other cell types[17,18,20,26] sigma-1 receptors are localized to focal adhesions in CHO-K1 cells in a sigma-1 receptor ligand dependent and F-actin stabilizing manner.

Interestingly, a 30 sec rinsing condition (before fixation) with DPBS with divalent cations present appeared to be a critical factor for retaining the sigma-1 receptors in the FAC. When the cells were rinsed with DMEM (without serum), instead of DPBS, the appearance of the sigma-1 receptors was also preserved in FAC, leading to the conclusion that the presence of divalent cations stabilized the sigma-1 receptors in the FAC, but did not appear to stimulate translocation of the sigma-1 receptors to the FAC.

One established function of sigma-1 receptors is modulation of the activity of Kv1.4 potassium channels, presumably as regulatory inhibitory subunits of these channels[11]. Under the experimental conditions reported in this paper the Kv1.4 potassium channels were confined to FAC. The sigma-1 receptor distributed to FAC, on the other hand, indicating a clear regulation by sigma-1 receptor ligands. Although the state of localization of Kv1.4 potassium channels was not assessed with regard to the sigma-1 receptor ligands in this study, it is reasonable to propose that increased focal adhesion targeting of the sigma-1 receptors is a regulatory mechanism for modulation of Kv1.4 voltage-gated channels in FAC in CHO-K1 cells. Volume-regulated chloride channels (VRCC) have also been reported to be profoundly inhibited by sigma-1 receptor ligands in various tumor cells[12]. Although little has been reported in the literature regarding the presence of ion channels in focal adhesions, a recent report is consistent with the presence of HERG potassium channels in the FACs[27].

Previous investigations have shown that in NG-108 cells treated with (+)-pentazocine, sigma-1 receptors translocated to the plasma membrane[18]. Our data further demonstrate that in CHO-K1 cells, the sigma-1 receptors target to the FAC via a specific drug response without a concomitant increase in the total focal adhesion contacts, as measured by the marker protein for FAC, talin (Fig. 3). Sigma-1 receptor ligands such as (+)-pentazocine[18], haloperidol and iodoazidococaine[28], may result in conformational changes in the receptor, leading to recruitment of the receptor to FAC via translocation pathways. It is also possible that multiple intracellular or extracellular signal transduction pathways are triggered by sigma-1 receptor ligands that indirectly result in FAC translocation of the sigma-1 receptors.

Interestingly, stabilization of F-Actin by phalloidin led to an increase in the level of talin and sigma-1 receptors together in FAC. Actin morphology remained intact after the sigma-1 receptor ligand treatments, whereas after phalloidin application, strong extended F-actin stress fibers formed (Fig. 3. H) with sigma-1 receptors localized to the tips of the strong fibers. The increase of sigma-1 receptors in FAC induced by the irreversible phalloidin F-actin polymerization indicated that the sigma-1 receptors are stabilized in FAC by F-actin polymerization.

FAC have been shown to interact in signaling pathways with lipid raft proteins, although it's not clear how lipid rafts commute between ER bundles and plasma membrane rafts[29]. Lisanti and coworkers showed that caveolin-1 and caveolin-2 proteins, which are enriched in lipid rafts in a phosphorylated form, localized to FAC, whereas unphosphorylated caveolins localized to the ER[29]. Previously it had been shown by Hayshi and Su that sigma-1 receptors associated with caveolin-2 in ER lipid rafts[30]. The mechanism by which the rate and direction of these processes are integrated with the cytosolic transport machinery remains obscure. There is also no clear evidence for how lipid droplets may be mobilized via actin filaments. In this regard there may well be an intimate relationship between lipid rafts and sigma-1 receptors regarding translocation to the barbed ends of actin filaments. Fluorescence recovery after photobleaching (FRAP) experiments performed in the Su laboratory suggested that the transfected GFP-sigma-1 receptors were able to translocate quickly throughout the entire network via the membranes of the ER[26].

The intracellular *in situ *sigma-1 receptor stability assessed by [^125^I]-IACoc photolabeling (Fig. 5.) is consistent with the possibility that recruitment of the sigma-1 receptors to FAC from intracellular pools occurs. In this regard it is further relevant that IACoc also increased appearance of the sigma-1 receptors in the FAC (Fig. 4.).

Further studies are necessary to establish the role and the level of sigma-1 receptor involvement in lipid rafts and determine the direct sigma-1 receptor/lipid raft trafficking pathway into the FAC and the plasma membrane.

## Methods

### Immunocytochemistry

Coverslips (Fisher #12-545-82) were precleaned overnight in Aqua Regia, rinsed three times with double distilled H_2_O and stored in 100% ethanol. On the day of seeding, coverslips were placed into each well of a 24 well cell culture cluster (Costar #3524), dried and coated w 0.01% poly-L-ornithine (Sigma #5666) for 30 min, rinsed three times with double distilled H_2_O for10 min each time, aspirated and dried under UV illumination.

CHO-K1 cells were split in the amount of 1,000 cells/coverslip and grown (in a 37°C incubator with 5% CO_2_) for 72 h in DMEM supplemented with 10% (vol/vol) of cosmic calf serum (HyClone #SH30087-03) and a 1× non-essential aminoacid solution (Sigma #M7145) to reach a well-spread morphology and confluence of 70–80%. Cells were briefly rinsed twice for 30 sec with 1× DPBS with or where indicated without CaCl_2 _and MgCl_2 _(100 μg/ml) and fixed with sterile-filtered 3.7% paraformaldehyde, 0.02% picric acid 1× DPBS, pH 7.4 for 15 min. After fixation, cells were rinsed three times with 1× PBS, quenched with 100 mM glycine 1× PBS for 3 min, rinsed three times with 1× PBS (each rinsing step was 3 min) and permeabilized with 0.1% Triton X-100 for 3 min. Blocking reagent (10% normal goat serum, Biomeda # ES1028) with 0.1% Triton X-100 present was used for 15 min. Incubation time with the primary antibody was for 1 h, and the cells were then rinsed three times with 1× PBS, 0.1% Triton X-100 and the secondary fluorophore conjugated goat-anti-rabbit/anti-mouse Fab (Molecular Probes # A11070 was used for Alexa 488 anti-rabbit or A11020 for Alexa594 anti-mouse conjugates). The solution was applied for 1 h (always in the presence of 2.5 μg/ml, 1× PBS, 0.1% Triton X-100). The cells were then rinsed three times with 1× PBS, 0.1% Triton, twice with PBS only, stained with 300 nM DAPI for two min, rinsed three times with PBS one min each time, aspirated and embedded into Mowiol mounting media in the presence of 2% Dabco, 2.5% Propyl Gallate and then fixed to the glass slide using clear nail polish (Electron Microscopy Sciences # 72180).

Images were taken under a Zeiss Axiovert 200 M epifluorescent microscope at 100× oil objective with Axiovision 4.3 Software. Morphometrical quantitation of the lengths of sigma-1 receptor and/or talin in the FAC was performed with the same software. The total amount of staining (in pixels) for each channel was measured for every image and divided by the amount of cells (determined by nuclear staining with DAPI). Phalloidin was from Molecular Probes (# A12381). Monoclonal antibodies against talin and Kv1.4 were from Sigma-Aldrich (#T3287 and K1264, respectively). The anti-sigma-1 receptor polyclonal antibody was prepared as described previously[23].

### Photolabeling *in situ *by [^125^I]-Iodoazidococaine in intact CHO-K1 cells

Synthesis of [^125^I]-Iodoazidicocaine was performed as described previously[21,22]. CHO-K1 cells were grown on two 10 cm Petri Dishes to 80% confluence. Cells were rinsed trice with DMEM (no serum) and 5 μM Haloperidol (as a protector) was added to one dish for 20 min. Then, the medium from the dishes was aspirated and 1 nM [^125^I]-Iodoazidicocaine was added into each dish in 1 ml of Dulbecco modified Eagles medium (DMEM) for 20 min. The cells were then rinsed three times with DMEM (30 sec each time) and once with DPBS, aspirated and dishes were exposed to an AH-6 mercury lamp for 5 sec at a distance of ~15 cm.

Cells were then rinsed three times with DPBS, aspirated, and 1 ml of Laemmli buffer 1× was added into each dish and incubated on ice for 20 min. Lysate was passed through a syringe with a 21 G needle (to shear DNA) and collected. 80 μl were loaded into each well for SDS-PAGE analysis.

To assess the stability of the photolabeled sigma-1 receptor, CHO-K1 cells were grown to 80% confluence on one 15 cm Petri Dish and photolabeled as described above. After exposure to light, cells were rinsed three times with DPBS (30 sec each time), briefly detached by addition of 0.05 Trypsine-EDTA, collected by a quick low spin and resuspended. Equal amounts of cells were split into 9 of 5 cm Petri Dishes in DMEM supplemented with 0.5% FBS. An additional aliquot (time zero sample) was collected by a brief centrifugation, the supernatant discarded and 350 μl of 1× Laemmli buffer was added to cells. An inhibitor of proteosomal degradation (Lactacystine 2 μM) and an inhibitor of lyzosomal degradation (Chloroquine 100 nM) were added to assess potential degradation pathways. For harvesting, the medium was removed from the dishes and 350 μl of 1× Laemmli buffer was added per dish, incubated for 20 min on ice, passed through a syringe with a 21 G needle and collected for loading onto the SDS gel. 80 μl of sample was loaded per well.

## Competing interests

The author(s) declare that they have no competing interests.
